# Clinical Characteristics and Long-Term Prognosis of Anti-LGI1 Encephalitis: A Single-Center Cohort Study in Beijing, China

**DOI:** 10.3389/fneur.2021.674368

**Published:** 2021-06-08

**Authors:** Tao-Ran Li, Yu-Di Zhang, Qun Wang, Xiao-Qiu Shao, Di-Yang Lyu, Rui-Juan Lv

**Affiliations:** ^1^Department of Neurology, Beijing Tiantan Hospital, Capital Medical University; China National Clinical Research Center for Neurological Diseases, Beijing, China; ^2^Department of Neurology, Xuanwu Hospital of Capital Medical University, Beijing, China; ^3^Department of Neurology, The Second Hospital of Hebei Medical University, Hebei Medical University, Shijiazhuang, China

**Keywords:** Lgi1, encephalitis, prognosis, relapse, follow-up

## Abstract

**Background:** This study aimed to analyze the clinical characteristics of anti-leucine-rich glioma-inactivated protein 1 (LGI1) encephalitis patients and investigate prognostic factors by using a large-sample and long-term follow-up cohort.

**Methods:** The clinical data of 45 patients (29 males; mean age, 57.0 years) from May 2014 to August 2019 were collected. All patients were followed up by face-to-face interviews in the third month after discharge and then by telephone and/or face-to-face interviews every 6 months until November 2020. We evaluated each patient's response to the initial treatments at the first interview and divided them into “responders” and “nonresponders.” Relapses were recorded. At the end of follow-up, each patient was evaluated and reclassified into “complete recovery” or “unhealed” groups. Intergroup differences were assessed.

**Results:** All patients presented with seizures at the initial consultation. Other common manifestations included cognitive dysfunction (82.2%), psychiatric disturbance (66.7%), sleep disorder (54.5%), and hyponatremia (66.7%). During the follow-up period (32.8 ± 13.5 months), six patients experienced relapse within 6–37 months. We observed that the patients who did not respond to the initial treatments and those who relapsed all had a poor long-term prognosis. The patients in the “unhealed” group were older (*p* = 0.009), had a lower incidence of generalized tonic–clonic seizures (*p* = 0.041), and had a higher probability of cerebrospinal fluid (CSF) abnormalities (*p* = 0.024) than those in the “complete recovery” group.

**Conclusion:** Anti-LGI1 encephalitis was characterized by seizures, cognitive impairment, psychiatric disturbance, and sleep disorders and was often accompanied by hyponatremia. Patients who responded poorly to the initial treatments and those patients who relapsed had dismal long-term prognoses. Advanced age and CSF abnormalities may be risk factors for poor prognosis, but these still need to be verified.

## Introduction

Leucine-rich glioma-inactivated protein 1 (LGI1) antibodies are probably the most common cause of limbic encephalitis and the second most common cause of autoimmune encephalitis after anti-N-methyl-D-aspartate receptor (NMDAR) encephalitis (1). The prominent clinical feature of anti-LGI1 encephalitis is drug-resistant epilepsy, but most patients respond well to first-line immunotherapy plus anti-epileptic drugs (AEDs) and can gradually discontinue medication after their condition has been relieved (1, 2). However, the disease can be very severe in the acute phase, and patients sometimes develop status epilepticus and require admission to the intensive care unit (ICU) (3–6) and may even die (6–8). Furthermore, as the research deepens, we have increasingly realized that the long-term prognosis of anti-LGI1 encephalitis may not be that good. Although most patients can acquire seizure freedom by immunotherapy, with or without AEDs (7, 9, 10), 28.0–66.7% of patients still have residual moderate to severe cognitive impairment (7, 9, 11), 21% reported persistent insomnia (7), and only 24–43% were able to return to work or to all premorbid activities (7, 11, 12). In addition, up to 77.8–88.9% of the patients developed visually detectable hippocampal atrophy in the long term (9, 13), and these patients tended to have poor memory recovery. Moreover, the previous viewpoint that anti-LGI1 encephalitis is a monophasic disorder has also been challenged; 12.5–35.3% of the patients will undergo at least one relapse after the acute phase (3, 6, 7, 11, 12, 14–17). Since the relapse interval can be as long as 98 months (7), the probability may be severely underestimated. On the other hand, some patients respond poorly to the initial treatments, including first-line immunotherapy. Patients who easily relapse and respond poorly to first-line immunotherapy may require early second-line immunotherapy (such as immunosuppressants), long-term immunotherapy, and AED treatment (1, 2).

Some studies have suggested that effective and long-term immunotherapy could improve the prognosis of anti-LGI1 encephalitis (9), while the complications of chronic immunotherapy cannot be ignored, as they occurred in approximately one-half of the patients (18). The benefits and risks of chronic immunotherapy and the duration of use should be carefully weighed, and it would be of great significance if we can predict which patients have a poor prognosis and/or are prone to relapse. Dong et al. found that conscious disturbance was a predictor of poor prognosis, but this conclusion was obtained in a mixed sample of autoimmune encephalitis patients with different etiologies, and the follow-up time was only 1 month (19). Previous studies on anti-NMDAR encephalitis patients have suggested that admission to the ICU, status epilepticus, delayed immunotherapy, and increased C-X-C motif chemokine 13 levels in cerebrospinal fluid (CSF) were all associated with poor outcomes (20–22). However, to the best of our knowledge, there is a paucity of large-sample and long-term follow-up studies assessing overall clinical prognosis in anti-LGI1 encephalitis.

Here, 45 anti-LGI1 encephalitis patients were enrolled from our tertiary epilepsy center, and they were all followed up for at least 12 months. With this study, we aimed to analyze their clinical characteristics and explore the risk factors for poor prognosis.

## Materials and Methods

### Patients

This study was reviewed and approved by the Ethics Committee of Beijing Tiantan Hospital and was in accordance with the Declaration of Helsinki. All patients gave written informed consent for participation and written consent to permit the publication of clinical details.

**Figure 1:**
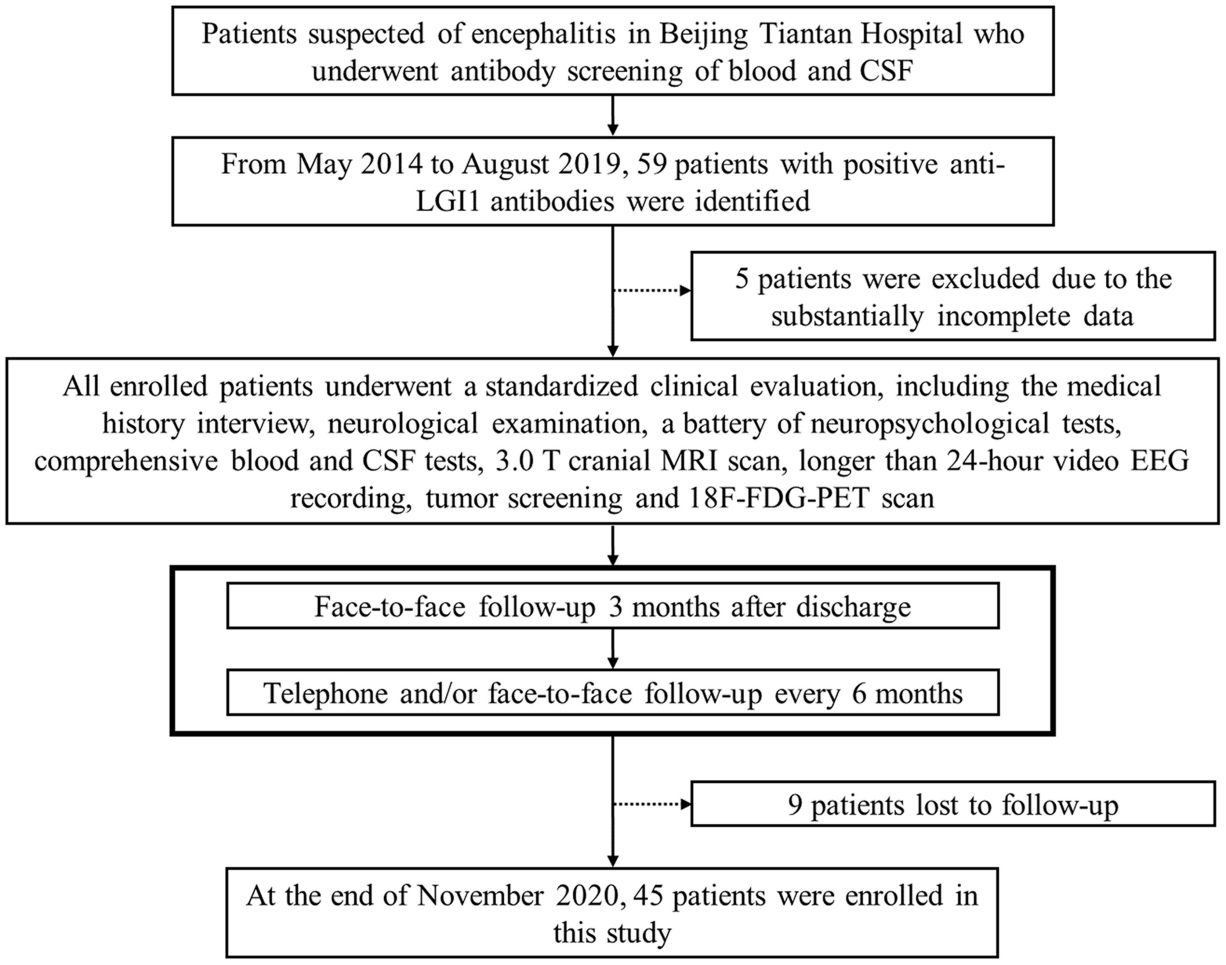
Flowchart of the patient selection process. CSF, cerebrospinal fluid; LGI1, leucine-rich glioma-inactivated protein 1; MRI, magnetic resonance imaging; EEG, electroencephalography; 18F-FDG-PET, 18-fluoro-deoxyglucose positron emission tomography.

According to the diagnostic consensus of the International Encephalitis Consortium (23), all patients suspected of encephalitis underwent antibody screening of blood and CSF. The serum and CSF samples were sent to the laboratory of neurological immunology of Peking Union Medical College Hospital for antibody testing. Commercially available fixed cell-based indirect immune-fluorescence biochips (Euroimmun AG, Lüebeck, Germany) were used to evaluate the serum and CSF titers for onconeural antibodies of anti-Hu, Yo, Ri, CV2/CRMP5, amphiphysin, Ma2/Ta, and the neuronal surface antibodies of anti-NMDAR, LGI1, contactin-associated protein-like 2, α-amino-3-hydroxy-5-methyl-4-isoxazole-propionic acid receptors 1 and 2, and γ-aminobutyric acid-B receptor. In all, 59 patients with positive anti-LGI1 antibodies from May 2014 to August 2019 in our tertiary epilepsy center were enrolled. In addition to the medical history interview and neurologic examination, the confirmed patients were also evaluated by a battery of auxiliary examinations, including comprehensive blood and CSF testing, 3.0 T cranial magnetic resonance imaging (MRI) scanning, longer than 24-h video electroencephalography (EEG) recording by using the 10–20 system of scalp electrode placement, neuropsychological testing [Montreal cognitive assessment (MoCA), Hamilton depression scale, Hamilton anxiety scale, and Pittsburgh sleep quality index], tumor screening, and 18-fluoro-deoxyglucose (^18^F-FDG) positron emission tomography (PET) scanning, which was acquired with a hybrid PET/computed tomography (CT) system (GE Healthcare, USA). The tumor screening consisted of chest CT scan, abdominal, thyroid, and pelvic ultrasounds, and tumor marker test. Clinical information was acquired by reviewing the patients' database records and charts. After discharge, to obtain measures of activities of daily living, cognitive functions, epileptic seizures, and other clinical information of the patients, we followed up with them by face-to-face interviews 3 months later, and subsequently, by telephone and/or face-to-face interviews every 6 months, until November 2020. Fourteen patients were excluded due to incomplete data or loss to follow-up. Details of the patient selection process are shown in a flowchart of the study (Figure 1).

### Prognostic Analysis

During the follow-up period, seizure freedom was defined as no clinical signs of seizures, which means that no seizures were observed, and there was no report of focal seizures (including auras) or generalized tonic–clonic seizures (GTCS) by the patients and/or informants; cognitive functions, mental states, and sleep quality were comprehensively assessed by interviews with the patients and/or informants, neurological examinations, and neuropsychological tests. Previous studies have suggested that both seizures and cognitive functions need to be improved for a certain period of time (11, 16). Thus, we evaluated each patient's response to the initial treatments, including immunotherapy and antiepileptic therapy, 3 months later; classification as a “responder” indicated that seizures were eliminated, and other clinical features were significantly improved and approaching (or returning) the state before the disease. A disease relapse was defined as symptoms reoccurring after stabilization or improvement for at least 3 months (9, 24). At the end of follow-up, each patient was comprehensively evaluated and reclassified into the “complete recovery” or “unhealed” group; “complete recovery” was defined as no sequelae and seizure freedom for at least 12 months, and “unhealed” was defined as having seizures or any sequelae.

### Statistics

The group comparisons of categorical variables and continuous variables were performed by using the chi-square test (or Fisher's exact test) and two-sample *t*-test, respectively. The relapse rate was estimated by using survival analysis. SPSS 22.0 (SPSS Inc., Chicago, IL, USA) was used for all analyses, and *p* < 0.05 was considered statistically significant.

## Results

### Clinical Features of All Participants

Clinical characteristics are summarized in Table 1, and details are provided below.

**Table 1:** Clinical characteristics of patients with anti-LGI1 encephalitis.

	**All (*n* = 45)**	**Complete recovery (*n* = 27)**	**Unhealed (*n* = 18)**	***p*-value**
Male	29 (64.4%)	17 (63.0%)	12 (66.7%)	0.799
Mean AOO, SD, range (years)	57.0, 12.9, 15.0–78.0	53.3, 13.9, 15.0–70.0	62.7, 9.0, 48.0–78.0	0.009
AOO <59 years	19 (42.2%)	13 (48.1%)	6 (33.3%)	0.324
Median diagnostic delay, SD, range (months)	4.1, 4.6, 0.3–24.0	4.7, 5.3, 0.3–24.0	3.3, 3.2, 0.5–12.0	0.330
Subacute ( ≤ 3 months) onset	29 (64.4%)	16 (59.3%)	13 (72.2%)	0.373
**Symptoms at diagnosis**				
Cognitive impairment	37 (82.2%)	22 (81.5%)	15 (83.3%)	1.000
Seizures	45 (100%)	27 (100%)	18 (100%)	1.000
FBDS	15 (33.3%)	7 (25.9%)	8 (44.4%)	0.197
Focal impaired awareness	25 (55.6%)	17 (63.0%)	8 (44.4%)	0.221
Focal aware	11 (24.4%)	6 (22.2%)	5 (27.8%)	0.732
GTCS	33 (73.3%)	23 (85.2%)	10 (55.6%)	0.041
Psychiatric disturbance	30 (66.7%)	15 (55.6%)	15 (83.3%)	0.053
Sleep disorders	24 (44Ava, 54.5%)	14 (26Ava, 53.8%)	10 (55.6%)	0.911
Hyponatremia	30 (66.7%)	16 (59.3%)	14 (77.8%)	0.197
Combined with other Abs	23 (42Ava, 54.8%)	13 (25Ava, 52.0%)	10 (17Ava, 58.8%)	0.663
MRI abnormalities	30 (66.7%)	18 (66.7%)	12 (66.7%)	1.000
^18^F-FDG-PET abnormalities	30 (33Ava, 90.9%)	17 (19Ava, 89.5%)	13 (14Ava, 92.9%)	1.000
**24-h video EEG**				
Abnormalities	40 (88.9%)	23 (85.2%)	17 (94.4%)	0.634
Typical rhythm evolution	20 (44.4%)	13 (48.1%)	7 (38.9%)	0.540
Subclinical seizures	11 (24.4%)	8 (29.6%)	3 (16.7%)	0.492
Interictal EEG abnormalities	40 (88.9%)	23 (85.2%)	17 (94.4%)	0.634
Slow waves	24 (53.3%)	15 (55.6%)	9 (50.0%)	0.714
Sharp/Spike waves	16 (35.6%)	8 (29.6%)	8 (44.4%)	0.309
**CSF**				
Abnormalities	20 (39Ava, 51.3%)	9 (23Ava, 33.3%)	11 (16Ava, 68.8%)	0.024
Cell count >5 cells/μl	3 (44Ava, 6.8%)	2 (26Ava, 7.7%)	1 (5.6%)	1.000
Protein >0.45 g/L	3 (44Ava, 6.8%)	1 (26Ava, 3.8%)	2 (11.1%)	1.000
OB	12 (40Ava, 30.0%)	4 (24Ava, 16.7%)	8 (16Ava, 50.0%)	0.037
Intrathecal IgG synthesis rate	7 (40Ava, 17.5%)	4 (24Ava, 16.7%)	3 (16Ava, 18.8%)	1.000
Anti-LGI1 Abs, serum	45 (45Ava, 100%)	27 (100%)	18 (100%)	1.000
Anti-LGI1 Abs, CSF	40 (44Ava, 90.9%)	22 (26Ava, 84.6%)	18 (100%)	0.133
Tumor	3 (6.7%)	2 (7.4%)	1 (5.6%)	1.000
Immunosuppressant	2 (4.4%)	0 (0%)	2 (11.1%)	0.155
GC + IVIG	18 (44Ava, 40.9%)	9 (26Ava, 34.6%)	9 (50.0%)	0.307
AEDs (one type or none)	32 (71.1%)	19 (70.4%)	13 (72.2%)	0.893
Median follow-up time, SD, range (months)	32.8, 13.5, 12.0–60.0	32.8, 12.8, 12.0–55.0	32.8, 14.9, 12.0–60.0	1.000

*Quantitative variables are expressed as the mean, SD, and range; categorical variables are reported as numbers and percentages of participants. The MRI abnormalities denoted abnormal signals and/or atrophy in the medial temporal lobe and/or basal ganglia; FDG-PET abnormalities referred to abnormal metabolism in the two regions. At the end of follow-up, each patient was evaluated comprehensively and reclassified into the “complete recovery” or “unhealed” group; “complete recovery” was defined as no sequela with seizure freedom maintained for at least 12 months, and “unhealed” was defined as having seizures or any sequelae. For the “p-value,” the statistical analysis was conducted by chi-square test or Fisher's exact test for categorical variables and independent-sample t-test for quantitative variables. Some patients had missing data. Hyponatremia means serum sodium <137 mmol/L*.

*LGI1, leucine-rich glioma-inactivated protein 1; AOO, age of onset; SD, standard deviation; FBDS, faciobrachial dystonic seizures; GTCS, generalized tonic-clonic seizures; Abs, antibodies; MRI, magnetic resonance imaging; 18F-FDG-PET, 18-fluoro-deoxyglucose positron emission tomography; EEG, electroencephalography; CSF, cerebrospinal fluid; OB, oligoclonal band; IgG, immunoglobulin G; IVIG, intravenous immunoglobulin; GC, glucocorticoid; AEDs, anti-epileptic drugs; Ava, available*.

**Figure 2:**
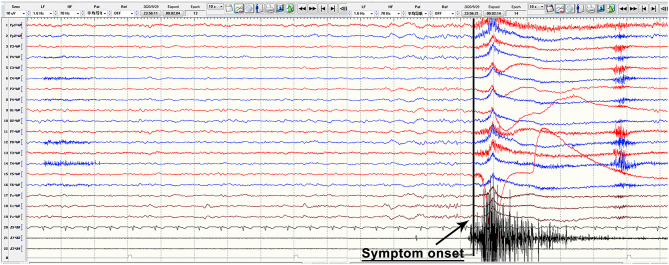
Typical ictal EEG manifestations of FBDS. The figure is a continuous EEG recording. During the recording, this anti-LGI1 encephalitis patient had FBDS attacks, which manifested as repeated twitching of the left upper arm and the left corner of the mouth, and electromyography showed a burst on the left side when the FBDS appeared, while EEG showed no rhythm changes and only obvious movement artifacts. EEG, electroencephalography; FBDS, faciobrachial dystonic seizures; LGI1, leucine-rich glioma-inactivated protein 1.

Anti-LGI1 antibodies were identified in 45 patients ranging in onset age from 15.0 to 78.0 years (mean: 57.0 ± 12.9 years), with a male to female ratio of 29 to 16. The mean time from symptom onset until diagnosis was 4.1 months (range: 0.25–24 months). All patients presented with seizures. More specifically, 15 (33.3%) patients had faciobrachial dystonic seizures (FBDS) with ictal EEGs that showed no rhythm changes when FBDS appeared (a typical EEG is shown in Figure 2); 25 (55.6%) patients had typical symptoms of seizures with focal impaired awareness, manifesting with dialeptic features accompanied by hand automatisms, lip smacking, vomiting sensation, fear sensation, or others, which occurred at a frequency of more than 1 and up to 20–30 times per day; 33 (73.3%) patients experienced GTCS, which tended to occur at night, with the total number ranging from 1 to 8, and 2 of these patients developed status epilepticus; and 11 (24.4%) patients had focal aware seizures, which were characterized by limb numbness, body shuddering, goosebumps, déjà vu, olfactory hallucinations, nausea, or others; in addition, 11 (24.4%) patients had subclinical attacks, whose EEG showed typical rhythm evolution, while no obvious behavioral changes were observed. Other common clinical manifestations included cognitive dysfunction (82.2%), psychiatric disturbance (66.7%), and sleep disorder (44 available, 54.5%). The average MoCA score was 16.9 ± 5.9 and ranged from 8.0 to 26.0. Among the 24 patients with sleep disorders, 16 (66.7%) had an increase in sleep, 5 (20.8%) experienced sleep deprivation, and 3 (12.5%) had behavioral abnormalities during sleep.

Of the 44 patients who underwent CSF examination, 3 had mildly increased protein concentrations (49.6–73.7 mg/dl), and 3 had mild white blood cell pleocytosis (13–16 cells/mm^3^). Additionally, 40 patients received additional CSF testing. We found that the intrathecal immunoglobulin G synthesis rate was increased (10.05–18.45 mg/d) in 7 patients, and an oligoclonal band (OB) was detected in 12 patients. Overall, 20 out of the 39 patients with complete CSF examinations had at least one of the abnormalities listed above. Anti-LGI1 antibodies were found both in the serum and CSF in 40 patients and only in the serum in five patients. Twenty-three (42 available, 54.8%) patients had other autoimmune antibodies, mainly anti-thyroglobulin antibodies, anti-thyroid peroxidase antibodies, or anti-nuclear antibodies. Thirty (66.7%) patients had hyponatremia (<137 mmol/L), and 13 of these patients (43.3%) showed stubborn conditions. Screening for malignant tumors revealed normal findings in most patients (42 available, 93.3%), with the exception of three subjects who had rectal adenoma, focal liver lesions, and lung cancer, respectively.

**Figure 3:**
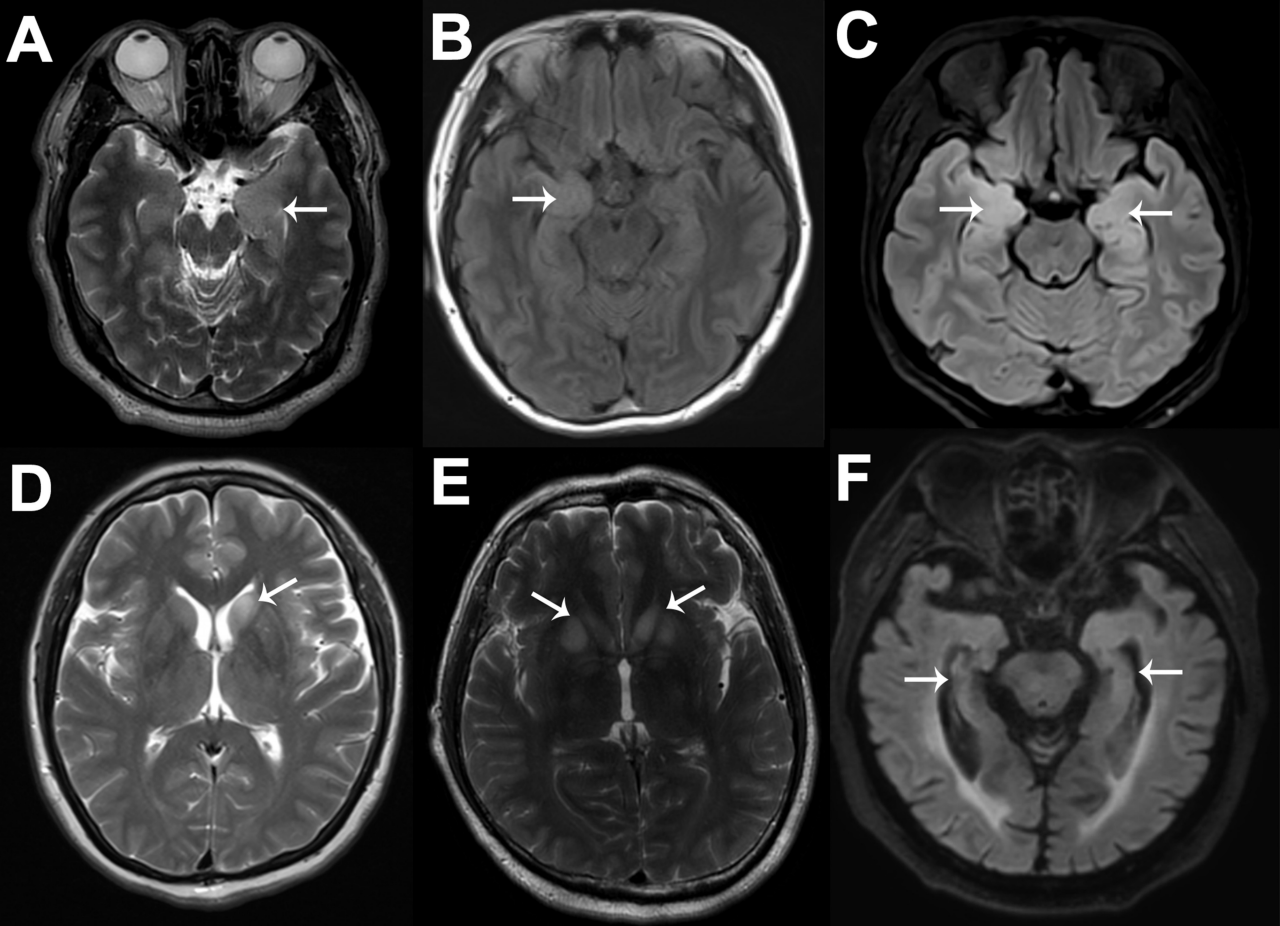
Typical MRI images of six anti-LGI1 encephalitis patients. The cranial MRI images of these anti-LGI1 encephalitis patients were not exactly the same. Six patients are illustrated here. As shown by the white arrows, increased signals on MRI fluid-attenuated inversion recovery or T2 sequences can be seen in the left MTL **(A)**, right MTL **(B)**, bilateral MTL **(C)**, left BA **(D)**, and bilateral BA **(E)**, and hippocampal atrophy can be seen **(F)**. MRI, magnetic resonance imaging; LGI1, leucine-rich glioma-inactivated protein 1; MTL, medial temporal lobe; BA, basal ganglia.

Cranial MRI scans were performed in all patients; among them, 15 (33.3%) patients showed normal or nonspecific changes, and 30 (66.7%) patients showed specific changes related to anti-LGI1 encephalitis. In detail, 5 (16.7%), 7 (23.3%), and 12 (40%) patients showed left, right, or bilateral, respectively, medial temporal lobe (MTL) increased signals on MRI fluid-attenuated inversion recovery or T2 sequences. Two (6.7%) patients showed abnormal signals in the left and bilateral basal ganglia (BA), while only one patient exhibited FBDS. Additionally, 1 (3.3%), 1 (3.3%), and 2 (6.7%) patients showed left, right, or bilateral hippocampal atrophy, respectively, and there were no significant differences in the disease course between them and the others (*p* = 0.728). Notably, the proportion of MTL abnormalities in patients with focal impaired awareness seizures (17/25, 68.0%) was not significantly higher than that in patients with other types of seizures (11/20, 55.0%) (*p* = 0.371). Typical MRI images are shown in Figure 3. Brain FDG-PET images were available for review in 33 patients: 3 (9.1%) patients were normal, and 4 (12.1%), 8 (24.2%), and 18 (54.5%) patients demonstrated abnormal metabolism in the MTL, BA, or both regions, respectively. These abnormalities were mainly hypermetabolism, although two patients showed hypometabolism, which manifested as hypometabolism in the left insular lobe, anterior temporal lobe, and hippocampus in one patient and as hypometabolism in the right hippocampus combined with hypermetabolism of the bilateral BA in the other patient. In addition, we found that eight patients presented with hypometabolism in multiple cortical regions. All patients underwent longer than 24-h video EEG examination, and 33 of them developed seizures during the examination; among them, 20 (60.6%) patients showed typical rhythm evolution, while no obvious changes were found in the remaining patients, and the corresponding clinical manifestations were FBDS and limb numbness. During the interictal phase, 24 (53.3%) patients exhibited slow waves, and 16 (35.6%) patients exhibited paroxysmal sharp/spike waves; these epileptiform waves were mainly centered in the temporal (87.5%) or frontal regions (43.8%). Overall, 40 patients (88.9%) had at least one kind of abnormality. Comparatively, brain FDG-PET was the most sensitive test in detecting abnormalities (90.9%), followed by EEG (88.9%, although the proportion showing typical rhythm evolution dropped this measure of sensitivity to 44.4%), and MRI (66.7%). Among the 33 patients who were evaluated by using both MRI and FDG-PET, 29 (87.9%) patients showed that FDG-PET was more sensitive in detecting lesions than MRI.

Forty-four of the 45 patients received immunotherapy: 14 received high-dose corticosteroids, 16 received high-dose corticosteroids combined with intravenous immunoglobulins, 10 received isolated intravenous immunoglobulins, 2 received intravenous immunoglobulins combined with oral prednisone, and 2 received intravenous immunoglobulins followed by oral immunosuppressants (either mycophenolate mofetil or azathioprine). There were 42 patients who took AEDs; among them, 29 took one type of AED, 10 took two types, and 3 took three types. The patient who did not receive immunotherapy took two types of AEDs. He responded well to the AEDs and had fully recovered by the end of the follow-up period (42 months).

### Prognostic Analysis

The response to the initial treatment of each patient was evaluated at the end of the third month of follow-up. Thirty-three patients were classified as “responders,” and the remaining patients were classified as “nonresponders.” As shown in Supplementary Table 1, the “responders” were younger (*p* = 0.031) and had a higher incidence of GTCS (*p* = 0.004) at admission than the “nonresponders.” Importantly, during the follow-up period (34.9 ± 16.0 months), these “nonresponders” never fully recovered and were subsequently classified as part of the “unhealed” group at the end of follow-up, and it should be noted that they were removed from the “relapse” analysis.

The mean follow-up time of all patients was 32.8 ± 13.5 months (range: 12.0–60.0 months). None of the patients died during the follow-up period, but 6 (13.3%) showed relapse at 6.0, 12.0, 12.0, 14.0, 16.0, and 37.0 months. The overall estimated relapse rate was 9.3% at 1 year and 23.0% at 3 years. As shown in Supplementary Table 2, compared with the patients without relapse, the patients with relapse did not present significant differences in any factors. After clinical relapse, they received first-line immunotherapy again, and the symptoms were relieved to a certain extent; however, they still had mild cognitive impairment and temporal lobe epilepsy seizures. Importantly, during the remaining follow-up period (12.5 ± 12.7 months), their symptoms (mainly seizures) were never completely relieved, and they were classified as being in the “unhealed” group at the end of the follow-up period.

The patients who responded to the initial treatment and had no relapse were classified as the “complete recovery” group (*n* = 27); they had complete relief of symptoms by the end of the follow-up period (32.8 ± 12.8 months). As shown in Table 1, the patients in the “unhealed” group showed an older age (*p* = 0.009), a lower incidence of GTCS (*p* = 0.041), a higher probability of CSF abnormalities [*p* = 0.024, mainly driven by the incidence of OB (*p* = 0.037)], and a higher incidence of psychiatric disturbance (the difference approached statistical significance, *p* = 0.053) at admission than the patients in the “complete recovery” group.

## Discussion

Based in a tertiary epilepsy center, this study retrospectively analyzed the clinical characteristics of 45 patients with anti-LGI1 encephalitis and followed them for an average of 32.8 months. The follow-up time in this study was relatively longer than previous studies (3, 6, 9, 10). We found that 13.3% of the patients had a relapse within 6–37 months, which was consistent with the rate of 12.5–35.3% in previous studies (3, 6, 7, 11, 12, 14–17), and the estimated 3-year relapse rate was 23.0%. It should be noted that this indicator is easily affected by the follow-up time, sample size, and evaluation methods. Importantly, we also observed that the patients who responded poorly to the initial treatment in the first 3 months and those patients who relapsed had dismal long-term prognoses at the end of the follow-up; therefore, it may be reasonable to give them intensive and long-term treatment, including immunosuppressants and AEDs.

In our cohort, the sex ratio and age structure of the anti-LGI1 encephalitis patients were both similar to previous studies; that is, it usually affects middle-aged or older people and is more common in males (25–29). Perhaps because all the patients were enrolled from an epilepsy center, all patients (100%) had seizures, although this proportion was similar to the 75–100% reported in previous studies (3, 6, 8, 11, 17, 25–29). Drug-resistant epilepsy is the most important core symptom of these patients. FBDS has been thought to be an indicative seizure type that frequently preceded the onset of limbic encephalitis (30), and the incidence in our study was 33.3%, consistent with some important studies with an incidence of 34–47% (7, 8, 17); however, some studies reported a higher incidence of 66.7–100% (3, 6, 26, 28, 29). In addition to seizures, cognitive dysfunction, psychiatric disturbances, and sleep disorders were relatively common clinical manifestations, which occurred in 82.2, 66.7, and 54.5% of the patients, respectively, and these results were comparable to previous studies that reported incidences of 64.3–100% (3, 6, 8, 11, 17, 25, 26, 28, 29), 33.3–73% (3, 6, 8, 17, 27–29), and 18.2–48% (3, 8, 11, 25, 26, 28), respectively. In terms of auxiliary examinations, the rates of CSF pleocytosis and protein elevation were both 6.8%, and similar results were obtained in previous studies, with rates of 3.7–19.4% and 12.5–23.1%, respectively (3, 11, 27, 29). In contrast, the frequency of OB and the rate of increased intrathecal IgG synthesis rate were both higher (30.0 and 17.5%, respectively), suggesting that they may be more sensitive than routine CSF examinations. The incidence of hyponatremia in the present study was also within the scope of previous studies (66.7 vs. 39–80%, respectively) (8, 11, 17, 25, 26, 28, 29); hyponatremia is often caused by inappropriate secretion of the antidiuretic hormone, which may be related to the expression of LGI1 in the hypothalamus and the kidney (31). Additionally, we found that 90.9% of the patients had typical abnormalities reflected in FDG-PET imaging, and 66.7% of the patients had MRI abnormalities, suggesting that the sensitivity of FDG-PET was much higher than that of structural MRI. In the longer than 24-h video EEG, the rate of abnormalities, which reached 88.9%, was mainly attributable to findings during the interictal period, and only 44.4% of the patients had a typical rhythmic evolution. A previous study obtained a similar order of sensitivity: FDG-PET (77%), MRI [mesial temporal sclerosis (48%), T2 mesial temporal hyperintensity (41%)], and EEG epileptiform activity (30%) (8). Brain MRI in limbic encephalitis patients often shows hippocampal swelling in the acute phase, and with the progression of disease, hippocampal atrophy gradually appears (1). We compared the disease duration of the patients with hippocampal atrophy and the other patients but did not find significant differences. This result may have been a result of the small sample size of our study. Patients with MTL abnormalities often exhibit focal impaired awareness seizures; thus, we compared the rate of MTL abnormalities in the patients with focal impaired awareness seizures and the patients with other types of seizures. Similarly, we did not find significant differences, which was perhaps the result of the small sample size. Furthermore, it should be noted that both the sample size and the evaluation method influence statistical results; for example, a previous study focused on aspects of sleep found that all 27 patients with anti-LGI1 encephalitis had sleep disorders (29), and this incidence was much higher than that in other reports and the present study.

The definition of relapse based on previous studies is confusing; more specifically, some studies did not provide a clear definition and simply described in the *Results* section that “relapse” can occur as soon as 1 month after initial treatments (3, 6, 12, 15), while other reports clearly stipulated that “relapse” occurs after a certain period of stabilization (9, 11, 24). Considering that improvements in symptoms take some time, for example, seizures needed to be absent for at least 3 months during follow-up (11, 16), we defined the manifestations of patients in the first 3 months as a response to the initial treatments and the manifestations after the first 3 months (at least 12 months in our study) as an outcome of long-term prognosis; we also defined relapse as including a stable period for at least 3 months. Currently, factors related to the prognosis of anti-LGI1 encephalitis are still lacking. Ariño et al. grouped patients based on their 2-year cognitive performance outcomes and found that failure to respond to first-line immunotherapy and clinical relapses were predictors of poor cognitive outcomes (11). By comparison, our evaluation of therapeutic effects was a comprehensive evaluation that mainly focused on epileptic seizures but also evaluated cognitive function, psychiatric disturbances, and sleep disorders. Coincidentally, although we did not use the regression model, we observed similar results: all of the patients who did not respond to the initial treatments and those who relapsed had poor long-term prognoses. In addition, we found some cross-sectional differences between groups. More specifically, elderly patients, patients who did not have GTCS, and patients who had CSF abnormalities (mainly OB) tended to have no treatment response and remain with complications. Some previous studies have also shown that advanced age usually portends a poor prognosis in acute encephalitis patients with mixed etiologies (32–37); however, there were no specific explanations provided in those studies. From our perspective, advanced age usually signifies an overall worse state, and elderly patients tend to suffer from some age-related diseases, such as osteoporosis and diabetes, which may influence the effectiveness of immunotherapy and AEDs. Elderly patients are also prone to develop hyponatremia, which leads to the limitation of the use of sodium channel blockers. All these factors may lead to a poor outcome. To the best of our knowledge, no studies have reported that GTCS is a protective or a risk factor regarding the prognosis of encephalitis; however, we found that the “complete recovery” group had a higher incidence of GTCS, which needs further verification. In anti-NMDAR encephalitis, de Montmollin et al. found that the increased white blood cells in CSF indicated good neurological outcomes, but the study was performed in patients requiring ICU admission (38); others found a correlation between abnormal CSF (only pleocytosis and increased protein levels) and poor outcomes based on univariate analyses, but this result barely reached significance with a *p*-value of 0.049, and the results of their multivariate regression analysis were not described (36); and other reports described that the OB-included CSF findings had no relationships with clinical outcomes in patients with anti-NMDAR encephalitis or anti-neuronal antibody-associated encephalitis (39, 40). The “unhealed” group of patients also had a higher incidence of psychiatric disturbance (*p* = 0.053) than the “complete recovery” group, however, there have been no studies focusing on this factor. A previous study observed that timely immunotherapy and combined immunotherapy (steroids and intravenous immunoglobulins) were both associated with a good prognosis (3), but the study included only 14 patients, and we did not observe these differences. In addition, patients who receive second-line immunotherapy usually have a higher relapse rate and a worse prognosis (2), but this may be related to “severity bias” and has limited clinical significance. Notably, a recent systematic review focused on identifying variables associated with prognosis in autoimmune encephalitis patients concluded that altered consciousness, ICU admission, and no use of immunotherapy were factors associated with poor prognosis in anti-NMDAR encephalitis, and the delay in immunotherapy contributed to a variety of worse outcomes for patients with different types of autoimmune encephalitis, while factors such as older age, the presence of status epilepticus, CSF abnormalities, and MRI changes were unlikely to have significant prognostic value (41). Although this study was not specific to anti-LGI1 encephalitis and the quality of the enrolled literature was heterogeneous, it still reminds us that we should cautiously interpret the intergroup differences here. Whether these differences reflect actual conditions or statistical results needs to be explored with a larger sample size and longer-term follow-up study in the future.

We acknowledge a number of limitations and future directions that should be taken. First, the small sample size limited the statistical power of our analysis. Second, the patients were from a single tertiary epilepsy center, leading to unavoidable selection bias; for example, all the patients had seizures as the main manifestation, and no patients were severe enough to require admission to the ICU. Third, we did not perform regression analysis for the following reasons: the difference between the sample size and the number of variables was too small, and the patients who responded poorly to the initial treatments and the patients who relapsed were all in the “unhealed” group, that is, none of these patients were in the “complete recovery” group, which makes the regression analysis difficult to perform. Considering these limitations, multicenter collaboration to include more patients is needed in the future.

In this study, we described the clinical characteristics of anti-LGI1 encephalitis. We found that it was a disorder dominated by middle-aged and elderly males, drug-resistant seizures were its most prominent symptoms, and its other primary symptoms included cognitive impairment, psychiatric disturbances, and sleep disorders. In addition, hyponatremia was a common feature, while the incidence of CSF abnormalities reflected in increased protein concentrations and white blood cell pleocytosis was low, and very few patients were found to suffer from tumors by cancer screening. Moreover, the sensitivity of FDG-PET was much higher than that of structural MRI and EEG. Although the disease was mostly curable and monophasic, some of the patients did have serious sequelae. We observed that the patients who did not respond to the initial treatments and those who relapsed all had a poor long-term prognosis. Thus, we suggest that these patients should receive intensive and long-lasting maintenance immunotherapy or second-line treatment as soon as possible. In addition, we also found that advanced age and CSF abnormalities may be associated with poor prognosis in anti-LGI1 encephalitis, however, these prognostic factors still need further confirmation.

## Data Availability Statement

The original contributions presented in the study are included in the article/Supplementary Material, further inquiries can be directed to the corresponding author.

## Ethics Statement

The studies involving human participants were reviewed and approved by the Medical Ethics Committee of Beijing Tiantan Hospital, Capital Medical University. The patients/participants provided their written informed consent to participate in this study.

## Author Contributions

R-JL, Y-DZ, QW, and X-QS provided the clinical data. T-RL and R-JL drafted the manuscript. T-RL and D-YL performed the statistical analyses. R-JL critically revised the manuscript for important intellectual content. All authors contributed to the article and approved the submitted version.

## Conflict of Interest

The authors declare that the research was conducted in the absence of any commercial or financial relationships that could be construed as a potential conflict of interest.
