# Supplementary material for: Clinical Characteristics and Long-Term Prognosis of Anti-LGI1 Encephalitis: A Single-Center Cohort Study in Beijing, China
Source: Front Neurol. 2021 Jun 8;12:674368. doi: 10.3389/fneur.2021.674368 (PMC8217831; doi:10.3389/fneur.2021.674368)
Supplement: Supplementary file 1 [file Data_Sheet_1.docx]

|  | Responders (n=33) | Nonresponders (n=12) | *p*-value |
| --- | --- | --- | --- |
| Male | 22 (66.7%) | 7 (58.3%) | 0.728 |
| Mean AOO, SD, range (y) | 54.6, 13.3, 15.0-76.0 | 63.9, 9.3, 49.0-78.0 | 0.031 |
| AOO <59 y | 15 (45.5%) | 4 (33.3%) | 0.467 |
| Median diagnostic delay, SD, range (m) | 4.4, 5.0, 0.3-24.0 | 3.4, 3.2, 0.5-12.0 | 0.551 |
| Subacute (≤3 m) onset | 21 (63.6%) | 8 (66.7%) | 1.000 |
| Symptoms at diagnosis |  |  |  |
| Cognitive impairment | 26 (78.8%) | 11 (91.7%) | 0.419 |
| Seizures | 33 (100%) | 12 (100%) | 1.000 |
| FBDS | 10 (30.3%) | 5 (41.7%) | 0.496 |
| Focal impaired awareness | 21 (63.6%) | 4 (33.3%) | 0.070 |
| Focal aware | 6 (18.2%) | 5 (41.7%) | 0.131 |
| GTCS | 28 (84.8%) | 5 (41.7%) | 0.004 |
| Psychiatric disturbance | 20 (60.6%) | 10 (83.3%) | 0.283 |
| Sleep disorders | 18 (32Ava, 56.3%) | 6 (50.0%) | 0.711 |
| Hyponatremia | 22 (66.7%) | 8 (66.7%) | 1.000 |
| Combined with other Abs | 17 (31Ava, 54.8%) | 6 (11Ava, 54.5%) | 1.000 |
| MRI abnormalities | 22 (66.7%) | 8 (66.7%) | 1.000 |
| ^18^F-FDG-PET abnormalities | 20 (23Ava, 87.0%) | 10 (10Ava, 100%) | 0.536 |
| 24-hour video EEG |  |  |  |
| Abnormalities | 28 (84.8%) | 12 (100%) | 0.303 |
| Typical rhythm evolution | 16 (48.5%) | 4 (33.3%) | 0.366 |
| Subclinical seizures | 9 (27.3%) | 2 (16.7%) | 0.699 |
| Interictal EEG abnormalities | 28 (84.8%) | 12 (100%) | 0.303 |
| Slow waves | 19 (57.6%) | 5 (41.7%) | 0.344 |
| Sharp/Spike waves | 9 (27.3%) | 7 (58.3%) | 0.080 |
| CSF |  |  |  |
| Abnormalities | 13 (28Ava, 46.4%) | 7 (11Ava, 63.6%) | 0.333 |
| Cell count >5 cells/μL | 2 (32Ava, 6.3%) | 1 (8.3%) | 1.000 |
| Protein >0.45 g/L | 2 (32Ava, 6.3%) | 1 (8.3%) | 1.000 |
| OB | 7 (29Ava, 24.1%) | 5 (11Ava, 45.5%) | 0.254 |
| Intrathecal IgG synthesis rate | 5 (29Ava, 17.2%) | 2 (11Ava, 18.2%) | 1.000 |
| Anti-LGI1 Abs, serum | 33 (100%) | 12 (100%) | 1.000 |
| Anti-LGI1 Abs, CSF | 28 (32Ava, 87.5%) | 12, 100% | 0.562 |
| Tumor | 2 (6.1%) | 1 (8.3%) | 1.000 |
| Immunosuppressant | 0 (0%) | 2 (16.7%) | 0.067 |
| GC+IVIG | 12 (32Ava, 37.5%) | 6 (50.0%) | 0.506 |
| AEDs (one type or none) | 22 (66.7%) | 10 (83.3%) | 0.460 |
| Median follow-up time, SD, range (m) | 32.1, 12.7, 12.0-55.0 | 34.9, 16.0, 12.0-60.0 | 0.537 |

Supplementary Table 1. Baseline comparisons between patients responded and non-responded to the initial treatments

Quantitative variables are expressed as the mean, SD and range; categorical variables are reported as numbers and percentages of participants. The MRI abnormalities denoted abnormal signals and/or atrophy in the medial temporal lobe and/or basal ganglia; FDG-PET abnormalities referred to abnormal metabolism in the two regions. Response to the initial treatment of each patient was evaluated at the end of the third month of follow-up, the “responders” meant that patients got rid of seizures, and other clinical features are significantly improved, approaching (or returning) to the state before the disease, and the “nonresponders” as having any sequela. For the “*p*-value”, the statistical analysis was conducted by chi-square test or Fisher’s exact test for categorical variables and independent-sample t-test for quantitative variables. Some patients had missing data. Hyponatremia means serum sodium less than 137 mmol/L.

Abbreviations: LGI1, leucine-rich glioma-inactivated protein 1; AOO, age of onset; SD, standard deviation; FBDS, faciobrachial dystonic seizures; GTCS, generalized tonic-clonic seizures; Abs, antibodies; MRI, magnetic resonance imaging; 18F-FDG-PET, 18-fluoro-deoxyglucose positron emission tomography; EEG, electroencephalography; CSF, cerebrospinal fluid; OB, oligoclonal band; IgG, immunoglobulin G; IVIG, intravenous immunoglobulin; GC, glucocorticoid; AEDs, anti-epileptic drugs; Ava, available.

|  | Relapse (n = 6) | Non-relapse (n = 27) | *p*-value |
| --- | --- | --- | --- |
| Male | 5 (83.3%) | 17 (63.0%) | 0.637 |
| Mean AOO, SD, range (y) | 60.5, 8.6, 50.0-76.0 | 53.3, 13.9, 15.0-70.0 | 0.233 |
| AOO <59 y | 2 (33.3%) | 13 (48.1%) | 0.665 |
| Median diagnostic delay, SD, range (m) | 3.0, 3.5, 1.0-10.0 | 4.7, 5.3, 0.3-24.0 | 0.474 |
| Subacute (≤3 m) onset | 5 (83.3%) | 16 (59.3%) | 0.379 |
| Symptoms at diagnosis |  |  |  |
| Cognitive impairment | 4 (66.7%) | 22 (81.5%) | 0.584 |
| Seizures | 6 (100%) | 27 (100%) | 1.000 |
| FBDS | 3 (50.0%) | 7 (25.9%) | 0.336 |
| Focal impaired awareness | 4 (66.7%) | 17 (63.0%) | 1.000 |
| Focal aware | 0 (0%) | 6 (22.2%) | 0.563 |
| GTCS | 5 (83.3%) | 23 (85.2%) | 1.000 |
| Psychiatric disturbance | 5 (83.3%) | 15 (55.6%) | 0.364 |
| Sleep disorders | 4 (66.7%) | 14 (26Ava, 53.8%) | 0.672 |
| Hyponatremia | 6 (100%) | 16 (59.3%) | 0.077 |
| Combined with other Abs | 4 (66.7%) | 13 (25Ava, 52.0%) | 0.664 |
| MRI abnormalities | 4 (66.7%) | 18 (66.7%) | 1.000 |
| ^18^F-FDG-PET abnormalities | 3 (4Ava, 75.0%) | 17 (19Ava, 89.5%) | 0.453 |
| 24-hour video EEG |  |  |  |
| Abnormalities | 6 (100%) | 23 (85.2%) | 1.000 |
| Typical rhythm evolution | 3 (50.0%) | 13 (48.1%) | 1.000 |
| Subclinical seizures | 1 (16.7%) | 8 (29.6%) | 1.000 |
| Interictal EEG abnormalities | 6 (100%) | 23 (85.2%) | 1.000 |
| Slow waves | 4 (66.7%) | 15 (55.6%) | 1.000 |
| Sharp/Spike waves | 1 (16.7%) | 8 (29.6%) | 1.000 |
| CSF |  |  |  |
| Abnormalities | 4 (5Ava, 80.0%) | 9 (23Ava, 39.1%) | 0.153 |
| Cell count >5 cells/μL | 0 (0%) | 2 (26Ava, 7.7%) | 1.000 |
| Protein >0.45 g/L | 1 (16.7%) | 1 (26Ava, 3.8%) | 0.345 |
| OB | 3 (5Ava, 60.0%) | 4 (24Ava, 16.7%) | 0.075 |
| Intrathecal IgG synthesis rate | 1 (5Ava, 20.0%) | 4 (24Ava, 16.7%) | 1.000 |
| Anti-LGI1 Abs, serum | 6 (100%) | 27 (100%) | 1.000 |
| Anti-LGI1 Abs, CSF | 6 (100%) | 22 (26Ava, 84.6%) | 0.566 |
| Tumor | 0 (0%) | 2 (7.4%) | 1.000 |
| Immunosuppressant | 0 (0%) | 0 (0%) | 1.000 |
| GC+IVIG | 3 (50.0%) | 9 (26Ava, 34.6%) | 0.647 |
| AEDs (one type or none) | 3 (50.0%) | 19 (70.4%) | 0.375 |
| Median follow-up time, SD, range (m) | 28.7, 12.6, 12.0-47.0 | 32.8, 12.8, 12.0-55.0 | 0.477 |

Supplementary Table 2. Baseline comparisons between patients with clinical “relapse” and “non-relapse”

Quantitative variables are expressed as the mean, SD and range; categorical variables are reported as numbers and percentages of participants. The MRI abnormalities denoted abnormal signals and/or atrophy in the medial temporal lobe and/or basal ganglia; FDG-PET abnormalities referred to abnormal metabolism in the two regions. A disease relapse was defined as the symptoms occurring again after stabilization or improvement for at least 3 months. For the “*p*-value”, the statistical analysis was conducted by chi-square test or Fisher’s exact test for categorical variables and independent-sample t-test for quantitative variables. Some patients had missing data. Hyponatremia means serum sodium less than 137 mmol/L.

Abbreviations: LGI1, leucine-rich glioma-inactivated protein 1; AOO, age of onset; SD, standard deviation; FBDS, faciobrachial dystonic seizures; GTCS, generalized tonic-clonic seizures; Abs, antibodies; MRI, magnetic resonance imaging; 18F-FDG-PET, 18-fluoro-deoxyglucose positron emission tomography; EEG, electroencephalography; CSF, cerebrospinal fluid; OB, oligoclonal band; IgG, immunoglobulin G; IVIG, intravenous immunoglobulin; GC, glucocorticoid; AEDs, anti-epileptic drugs; Ava, available.
